# High-Resolution Rainfall Estimation Using Ensemble Learning Techniques and Multisensor Data Integration

**DOI:** 10.3390/s24155030

**Published:** 2024-08-03

**Authors:** Maulana Putra, Mohammad Syamsu Rosid, Djati Handoko

**Affiliations:** Department of Physics, FMIPA Universitas Indonesia, Depok 16424, Indonesia; maulana.putra11@ui.ac.id (M.P.); syamsu.rosid@ui.ac.id (M.S.R.)

**Keywords:** rainfall, ensemble learning, multisensor

## Abstract

In Indonesia, the monitoring of rainfall requires an estimation system with a high resolution and wide spatial coverage because of the complexities of the rainfall patterns. This study built a rainfall estimation model for Indonesia through the integration of data from various instruments, namely, rain gauges, weather radars, and weather satellites. An ensemble learning technique, specifically, extreme gradient boosting (XGBoost), was applied to overcome the sparse data due to the limited number of rain gauge points, limited weather radar coverage, and imbalanced rain data. The model includes bias correction of the satellite data to increase the estimation accuracy. In addition, the data from several weather radars installed in Indonesia were also combined. This research handled rainfall estimates in various rain patterns in Indonesia, such as seasonal, equatorial, and local patterns, with a high temporal resolution, close to real time. The validation was carried out at six points, namely, Bandar Lampung, Banjarmasin, Pontianak, Deli Serdang, Gorontalo, and Biak. The research results show good estimation accuracy, with respective values of 0.89, 0.91, 0.89, 0.9, 0.92, and 0.9, and root mean square error (RMSE) values of 2.75 mm/h, 2.57 mm/h, 3.08 mm/h, 2.64 mm/h, 1.85 mm/h, and 2.48 mm/h. Our research highlights the potential of this model to accurately capture diverse rainfall patterns in Indonesia at high spatial and temporal scales.

## 1. Introduction

Indonesia is a tropical country with various complexities in its rainfall patterns [1,2,3,4]. Based on data processing and analysis over 30 years (1991–2020), it has been identified that the rainfall distribution patterns in the Indonesian region consist of 487 monsoonal, 178 equatorial, and 34 local types. This has a direct impact on many aspects of life, such as transportation, agriculture, water resource management, and disasters [5,6,7,8]. Moreover, extreme rainfall can cause significant impacts on the economic sector, infrastructure, and public safety [9,10]. However, at present, the information regarding rainfall in Indonesia has regrettably not reached a high resolution, and the geographical coverage is limited.

Rainfall measurements in Indonesia are generally performed using two methods: direct observation via a rain gauge and indirect estimation via remote sensing [11,12,13]. The measurements using rain gauges are accurate; however, the total surface area of rain gauges in Indonesia covers only 1.29 × 10^−11^ of the country’s total area. This inadequate gauge density results in inaccuracies when representing the total rainfall across an area [14]. The distribution of rain gauges in Indonesia also highlights a concerning disparity in gauge density. Papua Island, the eastern region of Indonesia, has fewer rain gauges even though the region has local rain patterns with its diverse topography. In contrast, Java Island, the southern region of Indonesia, has a monsoon pattern and non-complex topography, but the rain gauge distribution there is very dense. The optimum rain gauge network has been the subject of research and operation in Indonesia over the years [15,16]. However, the national weather radar network does not cover the entire observation area in Indonesia [17]. Several weather radars also cannot explain a single relationship between radar reflectivity and rainfall [18]. The variability in this relationship can certainly affect the accuracy of rainfall estimates using weather radars. Meanwhile, weather satellite images with global coverage have resolution limitations [19]. In measuring rainfall in Indonesia, the necessity for a wide spatial coverage with a high level of resolution is the main challenge [20,21]. This condition explains why the existence of only one data source is insufficient to fulfill Indonesia’s need for reliable rainfall data.

Indonesia’s national rainfall information product is provided by the Meteorology, Climatology, and Geophysics Agency (BMKG) via its official website at www.bmkg.go.id, accessed on 31 July 2024. The information displayed is limited to rain classification images covering Indonesia with a time resolution of 1 h and an accumulation of 24 h. Apart from that, a research institution in Indonesia, the National Innovation Research Agency (BRIN), has the Sadewa website at the address https://sadewa.brin.go.id/sadewabgr/ (accessed on 24 June 2024), which provides information on rainfall predictions with a time resolution of 1 hours resulting from modeling with a spatial resolution of 1 km. The results of previous studies also have limitations. The generated products are typically not in the form of numerical data but are mostly in the form of classifications [22,23,24], are not yet able to produce comprehensive spatial products, tend to be limited to data per point or region [25,26,27,28,29], and do not provide actual and up-to-date information to meet real-time needs [20,30,31,32,33]. Based on these facts, there is no numerical, spatial, high-resolution rainfall estimation model that uses observational equipment. In fact, information about rainfall in Indonesia is really needed because it has a direct impact on many aspects of life [5,6,7,8], such as agriculture, because rainfall affects agricultural productivity [34]; transportation, because it can be the cause of transportation accidents [35,36,37,38]; and disasters, because its impact is often associated with disasters such as floods and landslides [9,10,39,40].

Currently, the existing rainfall estimation models cannot detect hidden patterns or non-linear trends in rainfall data, which are important features for producing accurate rainfall information products [41]. The dynamics of the air in the atmosphere may be significantly non-linear; however, there are still discernable patterns in its movement. These simple patterns may possibly be manually completed; however, for big data and complex non-linear patterns, the patterns may be generated using machine learning [42]. The implementation of machine learning in the field of meteorology includes the use of algorithms capable of processing extensive data, such as observation network data, weather radars, satellites, and weather models [43,44,45]. Through processing more significant data and more complex analysis, machine learning can be developed into a more accurate rainfall estimation model [46,47]. Several approaches to rainfall estimation have been explored by different researchers, and the importance of a high spatial and temporal resolution for accurate estimation precision has been emphasized [48,49,50,51,52,53,54]. However, using machine learning requires managing several challenging issues, namely, the unbalancing of classes [55], such as a disproportionate number of instances without rain or deficient rainfall compared with instances with high rainfall, a large number of missing attributes [56] during the process in which the sensor collects data, and the need to work incrementally immediately after new data are available. Addressing these challenges is crucial for harnessing the full potential of machine learning, especially in rainfall estimation.

Tree-based machine learning is a popular approach in the world of machine learning. The development of tree-based algorithms in machine learning has experienced a significant evolution. The developments began with decision trees, which are a simple tree structure used for decision making [57]. The drawbacks of decision trees include their tendency to overfit data and their typically lower accuracy [58,59], often necessitating ensemble methods to tackle these issues. The solution to the overfitting problem in decision trees is “ensembling”, which involves assembling many weak trees together into a strong forest, making predictions from each of the trees, and voting to decide on the winning prediction. This is further developed into a random forest, which uses a large number of trees to increase the accuracy and overcome overfitting [60]. With the bagging technique, weak learners/decision trees are arranged in parallel, and so they do not learn from each other; therefore, they have limitations in terms of computation [61]. In 1996, Schapire developed a boosting technique called AdaBoost, an ensemble learning technique that gives more weight to data misclassified by the previous model [62]. In many applications, AdaBoost is sensitive to both noise and data outliers and is slow in processing because it involves all data samples in each iteration [63]. Furthermore, gradient boosting advances development through the introduction of the concept of gradients and iteratively updating the model to reduce prediction errors [64]. Finally, extreme gradient boosting (XGBoost) improves boosting by increasing efficiency and accuracy with regularization, overfitting management, and layered trees [65]. The strength of XGBoost is its scalability in all scenarios. The scalability of XGBoost is due to several important systems and algorithmic optimizations. One is a new tree-learning algorithm for handling sparse data [65]. Moreover, parallel and distributed computing makes the learning process faster [66]. XGBoost is now the preferred method for developing predictive models due to its remarkable accuracy, efficiency, and adaptability [67,68]. Recently, XGBoost has even dominated the applied machine learning domain and has won several Kaggle competitions [69].

Applying XGBoost in rainfall estimation and related meteorological phenomena has demonstrated its efficacy and accuracy in predictive modeling. XGBoost was utilized alongside rain gauges, radars, and satellite data, achieving a high correlation and low RMSE, highlighting its robustness in integrating diverse data sources [70]. Despite its effectiveness, that study focused on one city, which may not be generalized well to different geographic locations with varying climate conditions. Coupling XGBoost with the Bat algorithm to estimate evapotranspiration showed superior performance in arid regions compared with other models [71]. Additionally, utilizing XGBoost for precipitation nowcasting outperformed other methods, proving its superiority in real-time applications [72]. Finally, integrating XGBoost with another method further enhanced precipitation nowcasting, demonstrating significant improvements in detection probability and error reduction [73]. The existing literature and studies show that XGBoost can be computationally demanding, especially when dealing with large datasets and deep trees. Training such models requires significant computational resources, which might not be available on less capable hardware. This complexity extends to hyperparameter tuning, which necessitates extensive experimentation to achieve optimal performance [66,74].

Currently, the application of machine learning to estimate rainfall in Indonesia is increasing and developing [75]. This study aimed to estimate rainfall in Indonesia, which exhibits seasonal, equatorial, and local characteristics. Our proposed approach integrates data from different instrument sources (i.e., rain gauges, weather radars, and weather satellites) to produce high-resolution rainfall estimates. Furthermore, ensemble learning, XGBoost, was applied to anticipate the sparse data that may occur due to the limited number of rain gauge points, limited radar coverage, and rain data imbalance problems. Finally, a spatial rainfall estimation model was built with a temporal resolution close to real time. Therefore, it is hoped that this study can benefit all stakeholders who use rainfall information in Indonesia, especially the transportation sector, which needs actual and high-resolution rainfall information.

## 2. Materials and Methods

**Figure 1:**
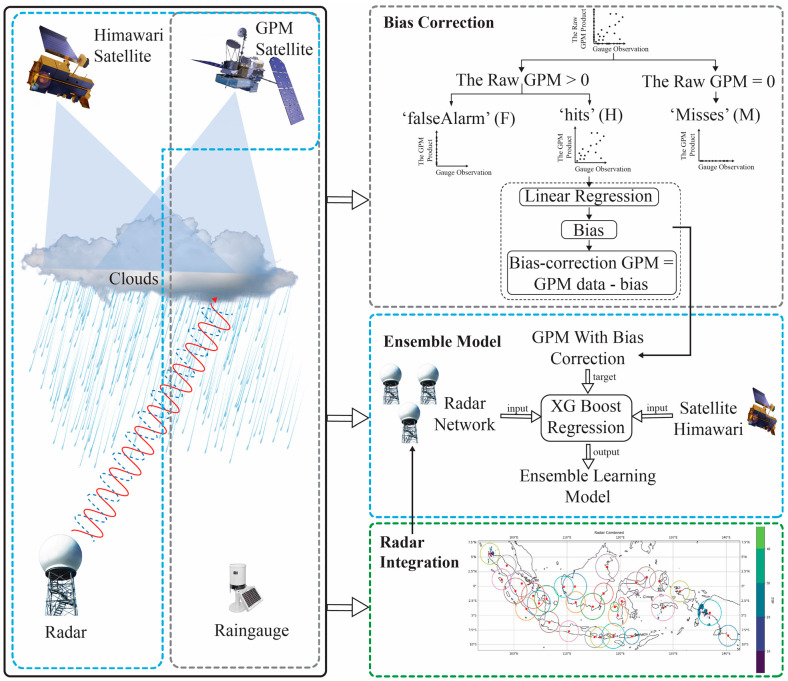
Architecture of the ensemble learning-based rainfall estimation model using multiple instruments: rain gauges, integrated weather radars, and weather satellites.

The data sources used in this research consisted of satellite data, weather radars, and rain gauges provided by the BMKG. The weather radar and rain gauge data were obtained directly from the BMKG monitoring system, which operates in various regions of Indonesia. A complex method for rainfall estimation is proposed in this study. This study included a bias correction process for satellite data to ensure data accuracy and weather radar data integration and implemented the XGBoost machine learning ensemble to process big data. The research stage diagram can be seen in Figure 1.

### 2.1. Study Area

**Figure 2:**
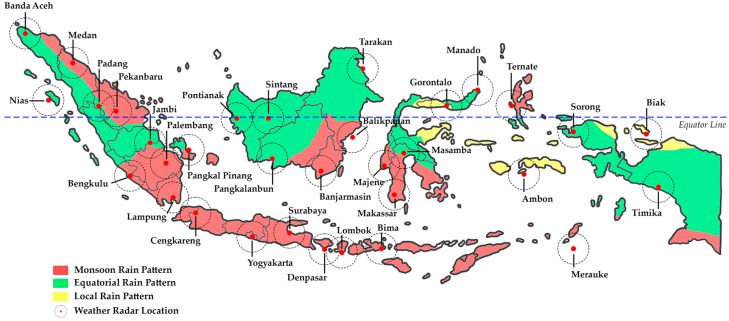
A map of the study area in Indonesia with three different rain patterns and a map of the weather radar network used in this research.

Indonesia is an archipelagic country situated in Southeast Asia, straddling the equator. Indonesia is located between 6° N to 11° S and 95° E to 141° E. Geographically, Indonesia consists of more than 17,000 large and small islands, making it the largest archipelagic country in the world. Overall, the land area is 1,993,662,036 km^2^; meanwhile, the water area reaches 6,653,341,439 km^2^ [76]. These geographic characteristics provide Indonesia with a diverse landscape, including mountains, tropical rainforests, and long beaches. More than 54,700 km of coastline connects the land with the sea, one of the main factors that influences the rainfall patterns in various regions of Indonesia. In this study, understanding the geography of Indonesia is fundamental to the process of rainfall estimation. Climatologically, Indonesia’s territory consists of seasonal zones and non-seasonal zones [77]. Indonesia’s territory is also divided into three types of rain patterns, namely, monsoonal, local, and equatorial types [78], as shown in Figure 2.

### 2.2. Data

In this rainfall estimation study, information from two different technologies, weather radar data and data from the Himawari satellite, became a training feature to obtain rainfall estimates. The main target of this estimation was global precipitation measurement (GPM) satellite data, which have been previously corrected using rain gauge data. This target was selected to ensure that the rainfall estimation results followed the results of the field observation measurements. The data period used covered the entire year of 2022. The training period spanned from January to November to maximize the training process. December was used for validation because it represents Indonesia’s peak rainy season, providing a range of conditions, from no rain to maximum rainfall.

Various satellite-based rainfall estimation products with a high spatial and temporal resolution have been developed to meet hydrometeorological data needs [79]. To date, the GPM-integrated multi-satellite retrievals (IMERG) product has shown more consistent performance and can be a good alternative for rainfall estimation [80,81]. The IMERG system operates in near-real time, providing data in two runs, known as early and late IMERG; subsequently, after the monthly gauge analysis is received, the final IMERG data are generated [82]. Early-run products have the potential for real-time applications with shorter delay times [83]. GPM combines the data from active and passive instruments within the GPM constellation to produce rainfall estimates, referred to as IMERG [84]. The GPM dataset is an early-run product produced every 30 min, covering 1 January to 31 December 2022. Spatial data with a pixel resolution of 10 km, covering a wide area of Indonesia, were used in this research.

A weather radar is an instrument that can detect various atmospheric parameters, such as rainfall, cloud movement, and wind speed, as well as the physical properties of rain or ice grains [85]. A weather radar transmits electromagnetic waves into the atmosphere, detects their reflections hitting objects, and measures the energy in radar reflectivity [86]. In this research, the resulting weather radar reflectivity data were used as a feature to estimate rainfall. In total, thirty-five weather radar units of various types were used to support this research. Of this number, six weather radar locations were used for data training, namely, the Lampung, Banjarmasin, Deli Serdang, Pontianak, Gorontalo, and Biak weather radars. The six radar locations used represent the existing rain patterns in Indonesia, which are seasonal, equatorial, and local rain patterns. The data are presented in a spatial resolution of 500 square meters. The temporal resolution of the weather radar data was updated every 10 min. The combination of a high spatial resolution and fast temporal resolution enabled very accurate and near-real-time rainfall estimates.

Another feature we used was data from the Himawari satellite in the form of brightness temperature. The Himawari satellite measures electromagnetic radiation emitted by various surfaces and the atmosphere in various spectral channels. These data produce brightness temperatures in various spectral channels, measured in kelvin (K). Equipped with an advanced Himawari imager (AHI) sensor, the Himawari satellite can produce various types of images. The Himawari satellite has 16 bands consisting of 3 visible bands, 3 near-infrared (NIR) bands, and 10 infrared (IR) or thermal bands. Each channel has a different wavelength, resolution, and use [87,88]. This research used IR band 13 with a wavelength of 10.4 μm. The selection of the IR channel was designed for use for observations at night since it can detect thermal radiation from objects on the Earth’s surface even though there is no direct sunlight available. In addition, the wavelength in band 13 is a clean longwave window with relatively high transparency toward electromagnetic radiation and is not significantly affected by water vapor in the atmosphere. This channel helps detect surfaces and clouds [88,89]. The spatial resolution of the Himawari satellite’s IR sensor is 2 km, indicating the sensor’s ability to distinguish objects at a minimum distance of that size.

The rainfall data were obtained from the Automated Weather Observing System (AWOS) equipment, which is an airport weather observation system that can provide weather information for 10 parameters, namely, wind speed, visibility, weather, sky conditions, temperature, relative humidity, wind chill, the heat index, pressure, and rainfall [90,91]. A rain gauge, which is one of the sensors in the AWOS equipment, is capable of measuring rain to an accuracy of 0.1 mm, so it is susceptible to detecting rainfall. With a time resolution of 1 min, this sensor can provide very detailed data about rainfall patterns in short intervals. With six rain gauge points, their use was advantageous in the GPM data correction process, which involved identifying rain events. Using the high-resolution rain gauges in the AWOS equipment increased the accuracy and precision of the rainfall data used in this research.

The instruments used in this study, including information on time resolution, spatial resolution, working principles, and units of product produced, are presented in Table 1.

### 2.3. Data Preprocessing

The data and instruments used in this study had different resolutions and units of measurement. The rainfall amount acquired from the rain gauges (mm), which were used to verify the GPM satellite rainfall product, was converted into the rain rate (mm/h) by taking into account the duration of the rainfall that occurred [92]. Rainfall data measured every 1 min by a rain gauge were converted into the rainfall intensity in units of millimeters per hour (mm/h). This conversion process was performed by measuring the total amount of rainfall every 10 min and then calculating the average rainfall intensity. In this way, rain gauge rainfall data measured every 1 min could be converted into the rainfall intensity displayed every 10 min in millimeters per hour.

The spatial resolution between the GPM satellite, Himawari satellite, and weather radars was another element that must be equalized. The weather radar pixels and Himawari satellites were adjusted to the GPM satellite pixels. We used bilinear interpolation techniques for the inter-pixel adjustments. In this technique, the new pixel value was determined based on the average by giving weight to the closest pixels. This technique was used because it can produce continuous data between known data points, making it easier to maintain spatial consistency. The bilinear interpolation process was carried out alternately on one of the vertical or horizontal sides [93]. Interpolation only involves the four nearest neighboring points, so the interpolation process is fast even when using a large dataset. By using the average value of the four nearest neighboring points, bilinear interpolation minimizes the loss of information that may occur [94].

The frequency of re-recording of all research instruments was equalized over a certain period. The temporal resolution must have the same interval [95]. The rain gauge data recorded every 1 min were downscaled to 10 min intervals to match the temporal resolution of the weather radars and Himawari satellites. The method used was to take a 10 min average of 10 consecutive data points. The data from the weather radars and Himawari satellites recorded every 10 min were maintained because they were in accordance with the desired resolution. For the training process, data that overlapped with the GPM data were used, and they were recorded every 30 min. The running process still used data every 10 min. Data from the GPM satellite recorded every 30 min were used for the training process.

Some of the data-filtering techniques applied in this research were resampling techniques for time series and intersection data. The resampling technique changed the time–frequency of data from one interval to another. With different sources and different time–frequencies, resampling helped make the data consistent and allowed for easier comparisons. Meanwhile, intersection was used to obtain the same elements between two or more data sets [96]. Intersection performed the operational function of merging several intersections of data sets that were previously incomplete to become sequentially complete. The research files were processed in the Zarr format, which stores each piece of data separately using data compression techniques to help reduce storage space requirements and speed up data transfer [97].

### 2.4. Bias Correction Strategy

The GPM product had several uncertainty sources, such as sensor calibration, retrieval errors, and orographic effects [98,99]; therefore, the product needed to be corrected for actual rainfall data from the rain gauges [100,101]. The bias correction method modified the bias correction strategy used in [102]. The first step in this research stage was to identify the rain events. In this context, only ‘hit’ events underwent bias correction; meanwhile, the other conditions were ignored in the analysis. A ‘hit’ event was when the GPM correctly detected rain. For the ‘hit’ events that were identified, corrections were made to reduce the bias between the GPM data and actual rain events that occurred using the linear regression method, which was previously performed by the authors of [103,104,105]. As previously explained in the data subsection, the six rain gauge points that were used for GPM correction are listed in Table 2. The bias correction could be carried out with at least one rain gauge available in each area to be corrected [106].

### 2.5. Integration of Weather Radar Data

A composite map can show the spatial rainfall distribution over a larger area, which is very important for understanding the current weather conditions and possibly predicting the rainfall cloud movements [17]. The number of weather radars scattered in the research area allowed for the intersection of coverage areas. To generate a composite map from several weather radars, the merging of the CMAX reflectivity values in each intersection coverage area was performed [107]. The complete information on the location, frequency band, and weather radar polarization used in this study is shown in Table 3.

### 2.6. Ensemble Learning Approach

Ensemble learning techniques have achieved state-of-the-art performance in diverse machine learning applications through the combination of predictions from two or more base models. We used the XGBoost algorithm for this research, more specifically, the XGBoost Python Package. XGBoost is a decision tree-based optimization technique that builds on the gradient descent method. The gradient descent method is used to optimize the loss function; additionally, regularization parameters are employed to prevent overfitting. The fundamental concept underlying the XGBoost algorithm is the minimization of the following objective function, which consists of the loss function and regularization terms [65]. Additionally, XGBoost is efficient in handling large datasets [108]. First, the procedure used in this algorithm involves dividing the original dataset into multiple sub-datasets. Then, each subset is randomly assigned to the base learner for prediction. The algorithm calculates the result of the weak learner based on a certain weight. Finally, the model results can be expressed as the weighted sum of the predicted results of all the decision trees.

Hyperparameters were used to enhance the algorithm’s performance results, significantly affecting various model tests. In this study, the hyperparameter method that was applied was Bayesian optimization. This method uses a probabilistic model to direct the search for the best hyperparameters. The previous search results are used to estimate the combination of hyperparameters that is most likely to provide better performance. The Bayesian optimization process consists of two main components: a surrogate model, which fits each observation to a target function, and an acquisition function, which balances exploitation and exploration to determine the next evaluation point. Bayesian optimization stabilizes exploration and exploitation to find optimal options while avoiding missing out on better configurations in areas that have not been evaluated [109,110,111].

The parameters that were optimized and expected to improve the model’s performance included seven parameters [110,112,113], as shown in Table 4 below.

For several reasons, Bayesian optimization has shown significant advantages in hyperparameter tuning for XGBoost compared with other methods. Primarily, it offers a systematic approach to exploring the hyperparameter space efficiently, utilizing prior information to guide the search process and update beliefs about the best regions to explore as more data are gathered. This probabilistic framework allows for a more informed and directed search, often resulting in faster convergence to optimal hyperparameters. For instance, studies such as that by Qiu et al. (2022) demonstrated that Bayesian optimization could achieve lower prediction errors and higher model performance with fewer iterations than traditional grid or random search methods. Moreover, Bayesian optimization effectively balances exploration and exploitation, enhancing the model’s accuracy and robustness [109,110,111,112,113,114].

### 2.7. Evaluation of Estimation Results

The estimated rainfall results from the ensemble learning model were evaluated using statistical and detection indicators [115]. The statistical indicators assessed the root-mean-square error (RMSE) values of the estimated rainfall; meanwhile, the detection indicators gauged the accuracy of the rainfall detection. The RMSE was used to measure the average magnitude of the errors between the estimated values and the actual values. The RMSE calculated the square root of the average of the squared differences between the predicted values and the actual values. The RMSE value provides an overall picture of the magnitude of the errors and can be useful in comparing model performance. In this case, accuracy refers to the extent to which the model could correctly classify rain occurrences or non-rain occurrences.

## 3. Results and Discussion

### 3.1. Data Correlation

The first task was to display and discuss the relationship between the input and target data that were used to build the rainfall estimation model. Specifically, we examined the relationship between the rainfall data produced by the GPM satellite and the rainfall data measured using the rain gauges.

**Figure 3:**
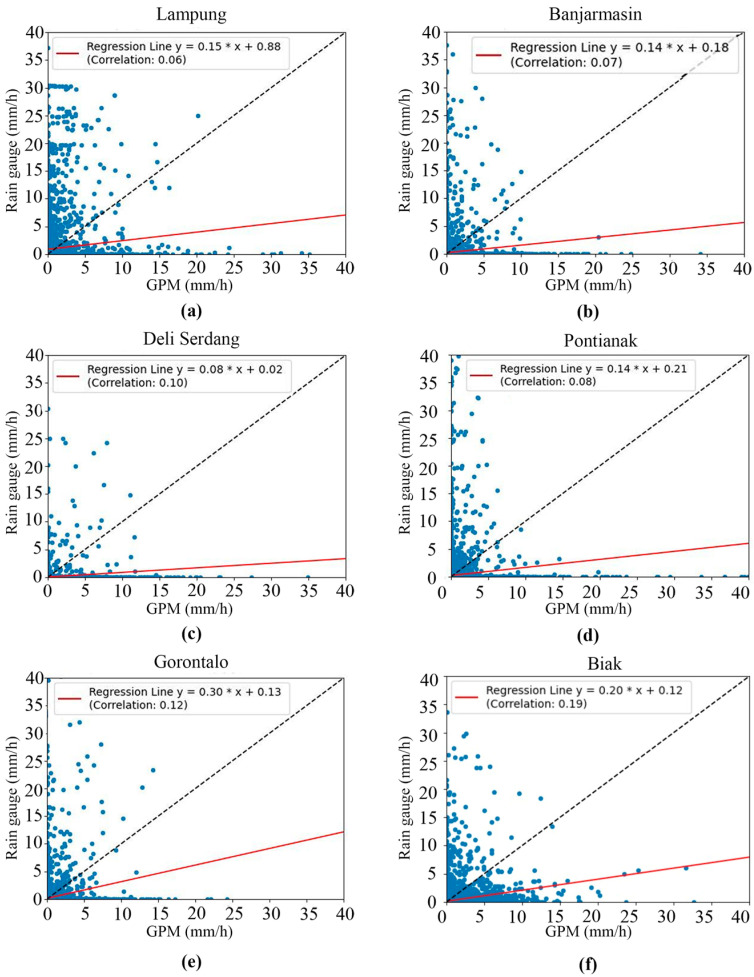
Scatter plots and correlation of GPM and rain gauge data in (**a**) Lampung, (**b**) Banjarmasin, (**c**) Deli Serdang, (**d**) Pontianak, (**e**) Gorontalo, and (**f**) Biak.

The rainfall estimates produced by the GPM satellite with rain-gauge-measured rainfall data revealed a low correlation [116,117], as shown in Figure 3. This may be the result of several errors in the data retrieval algorithms and sensors [118]. Other studies have stated that this discrepancy can be caused by cloud characteristics, climate, season, geographic location, and topography [119,120]. However, seeing the consistently low correlation in several locations, we suspect that the differences occurred due to lag time [121], i.e., the time delay between raindrops from clouds and rainfall on the Earth’s surface. This time delay means that when the GPM satellite observed rain from clouds, there was a certain time required for the raindrops to reach the Earth’s surface. Many factors, such as the distance between the cloud and the surface, can cause this delay. Therefore, the rainfall estimation data needed to be evaluated and corrected for bias.

**Figure 4:**
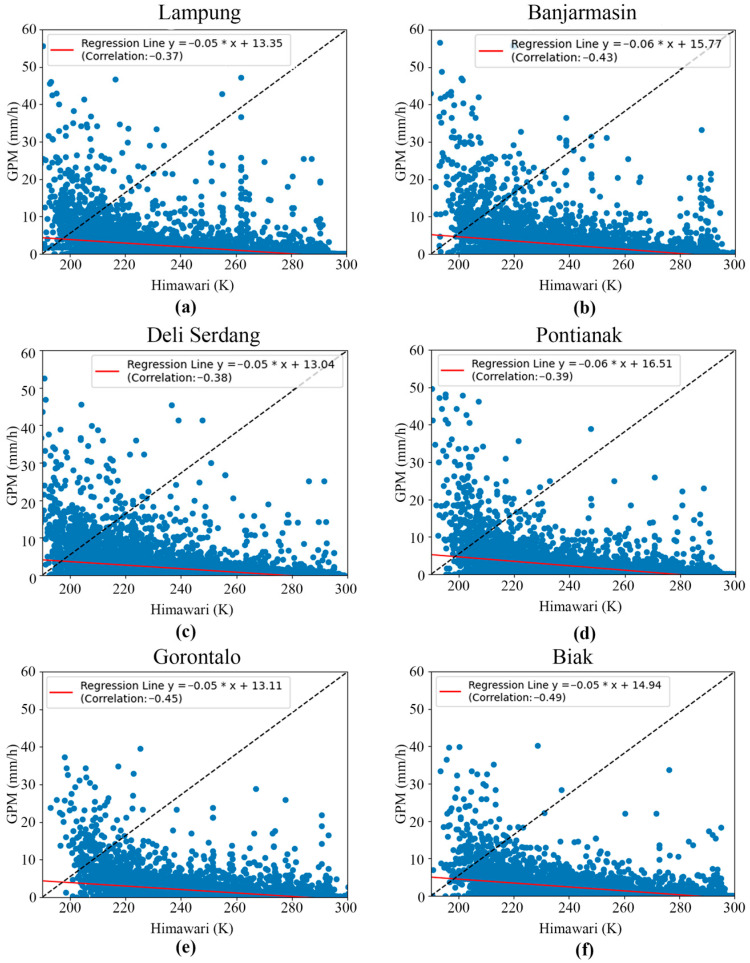
Scatter plots and correlations between GPM and Himawari satellite data in (**a**) Lampung, (**b**) Banjarmasin, (**c**) Deli Serdang, (**d**) Pontianak, (**e**) Gorontalo, and (**f**) Biak.

The second data correlation occurred between the rainfall data produced by the GPM satellite and the brightness temperature data measured by the Himawari satellite. The plotting of the two datasets is shown in Figure 4. Plotting the data at six points shows an inverse relationship between the cloud brightness temperature from the Himawari satellite data and the rainfall from the GPM satellite data, with a correlation value between −0.37 and −0.49. The correlation slope, even though insignificant, illustrates that when the cloud brightness temperature was low, the rainfall was high. Conversely, when the cloud brightness temperature was high, the rainfall was low. When the cloud brightness temperature decreases or becomes colder, this indicates that the cloud is thicker and tends to have larger water particles. The correlation value between the GPM satellite and the Himawari satellite was low due to differences in the working principles regarding the measurement targets. Working at a wavelength from 0.46 to 13.3 μm [122], the Himawari satellite can identify cloud droplets with a diameter of < 50 μm [123]. However, the presence of droplets in clouds does not always indicate rain. The droplets can turn into raindrops when small droplets collide with each other and merge through a process called coalition and coalescence [124]. However, not all droplets undergo this process until they reach a size large enough to fall as rain. In this case, the Himawari satellite is too sensitive for the task of identifying raindrops. Meanwhile, the GPM satellite has technical specifications that allow it to detect precipitation with a minimum size of 0.2 mm [125]. Therefore, the GPM satellite tends to be more accurate because it focuses on raindrops as the measurement object. Physically, this difference in measurement principles can result in a low correlation between the two satellites. The GPM satellite essentially measures raindrops, while the Himawari satellite measures cloud droplets, which do not necessarily turn into raindrops.

**Figure 5:**
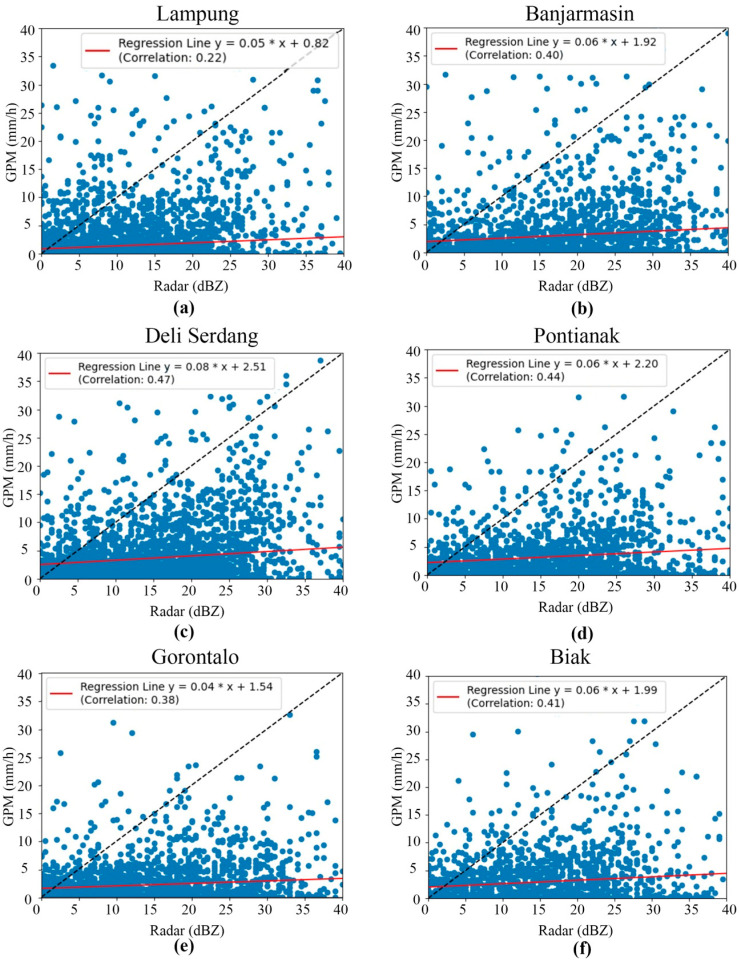
Scatter plots and correlations between GPM and weather radar data in (**a**) Lampung, (**b**) Banjarmasin, (**c**) Deli Serdang, (**d**) Pontianak, (**e**) Gorontalo, and (**f**) Biak.

There was a further correlation between the GPM satellite data and weather radar data. In physical terms, the higher the radar reflectivity (the dBZ value), the greater the possibility of high-intensity rain in that area. However, as presented in Figure 5, the data show that the correlation of the rainfall data produced by the GPM satellite with the radar reflectivity data was low. At a shorter measurement time resolution of 30 min, the correlation of the GPM satellite with the weather radars was lower than the daily or monthly measurement times [126]. This was due to the limitations of the early-run IMERG in capturing rapid changes in rain patterns. Topography was also a general factor that influenced the correlation of the GPM satellite with the weather radars [127]. The fundamental issue was the difference in the working principles of these two instruments. GPM identified precipitation directly via a combination of dual-frequency precipitation radar technology and the GPM microwave imager [128]. Meanwhile, the radars measured the reflectivity that was reflected back from the collection of droplets detected in the radar volume, but not from each droplet individually. High reflectivity can be caused by a few large droplets or many small droplets; therefore, it is difficult to determine the distribution and size categories of droplets based solely on reflectivity values [129,130,131]. In physical terms, the correlation between the GPM satellite data and the weather radars was low because GPM measured raindrops, while the radars measured a collection of droplets that may not necessarily be raindrops.

### 3.2. Bias Correction Result

Compared with the original product, the bias-corrected GPM product showed a better performance in estimating rainfall values. We display a graph of the probability density function (PDF), which is used to describe the probability distribution of a continuous random variable at a certain point. Additionally, a cumulative distribution function (CDF) graph is presented to illustrate the cumulative PDF of a random variable. This graph shows how often a random variable takes either a certain value or less than a certain value in its distribution; as a result, the graph can help in understanding the distribution, evaluating the probabilities, and identifying the patterns in GPM data before and after bias correction.

**Figure 6:**
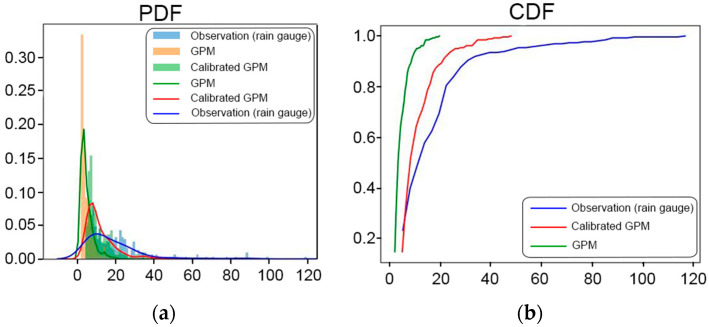
Comparison of (**a**) PDF and (**b**) CDF graphs between measured rainfall, uncorrected GPM, and corrected GPM. The *x*-axis represents the possible amount of rainfall. The *y*-axis represents the probability value.

The PDF and CDF graphs show a significant gap between the uncorrected GPM graph and the measured rainfall graph. This gap indicates that the initial satellite rainfall estimates had a significant bias; therefore, bias correction needed to be applied to bring the estimated results closer to the actual observation data. The GPM, which was not corrected, appeared to underestimate the measured rainfall. Meanwhile, the corrected GPM graph coincides more closely with the measured rainfall graph. This indicates that the applied bias correction succeeded in accurately approximating the observational data. The data distribution is shown in Figure 6.

### 3.3. Weather Radar Network

**Figure 7:**
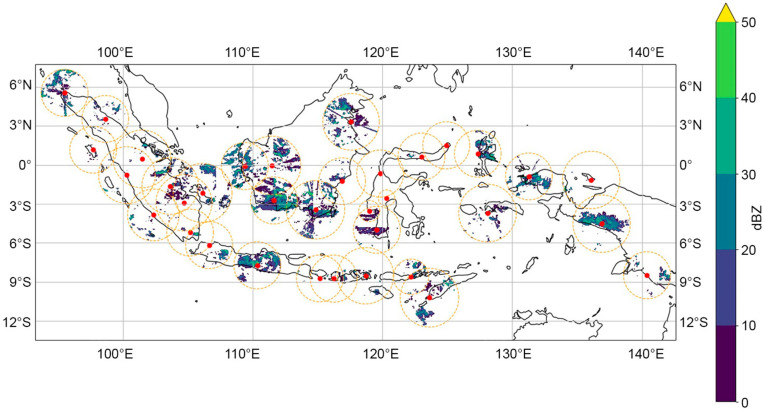
Data fusion technique for multiple weather radars with frequency, polarization, and coverage range differences. There are several coverage overlaps of the weather radars with the data from each radar, which are combined into a single data output. The dot is the weather radar location, and the circle is the weather radar range.

A national weather radar network was generated in this study with a total of 35 weather radars being integrated. This merging employed the spatial averaging of composite data, accommodating differences in frequency bands, such as C and X; differences in polarization, including single and dual; and differences in range, as illustrated in Figure 7.

### 3.4. Hyperparameter Tuning Results

Hyperparameter tuning optimization produced optimal values for each parameter using the Bayesian optimization method, as shown in Table 5.

**Figure 8:**
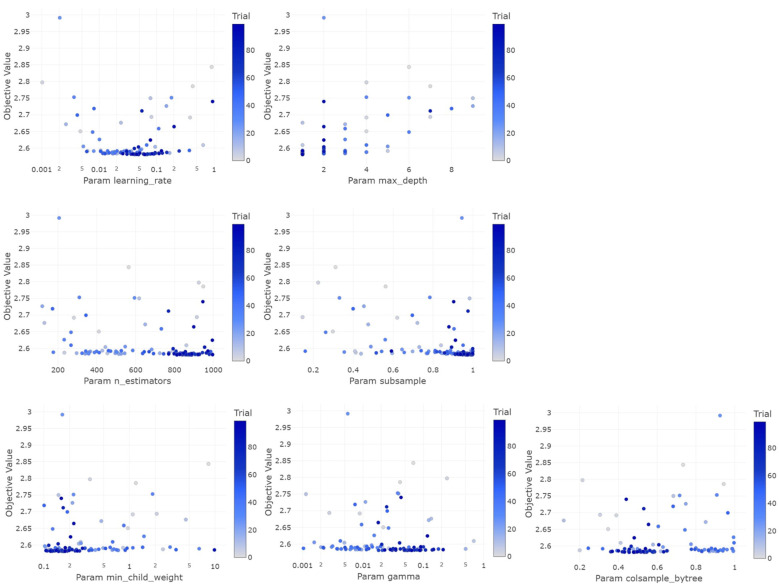
XGBoost hyperparameter optimization graph using Bayesian optimization.

Each hyperparameter was optimized to achieve a low objective value, which indicated a better model performance, as seen in Figure 8. The objective function in XGBoost regression is an evaluation metric, such as the mean squared error (MSE). In the learning_rate graph, it can be seen that the optimal value was in the range of around 0.01 to 0.1. A learning rate that is too high (>0.1) tends to produce a higher objective value, indicating overfitting. Meanwhile, a learning rate that is too low (<0.01) also does not provide the best performance because the model may be too slow to converge. For the hyperparameter max_depth, the optimal value was one. A too-deep depth (>6) is not optimal because it makes the model too complex. The graph for n_estimators shows that underestimating the number of estimators gave the best objective value. There is an ideal number of decision trees that should be used to achieve a balance between bias and variance. In the subsample graph, the optimal value was 0.96. For min_child_weight, the optimal value was 0.14. Higher values (>5) did not provide the best performance, indicating that the model requires some complexity to capture variations in the data. The gamma graph shows that the optimal value was 0.08, which shows that proper regularization is necessary to avoid overfitting. Finally, for colsample_bytree, the optimal value was around 0.4. Low or high values did not perform best, indicating that proper column sampling is necessary to reduce the correlation between trees.

### 3.5. Product and Evaluation

**Figure 9:**
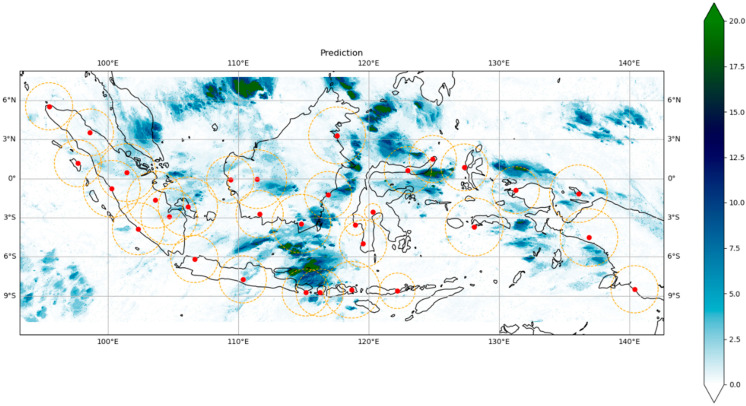
Rainfall estimation products in mm/h using ensemble learning techniques and multisensor data integration produced in this study.

After undergoing preprocessing and several processing steps, including weather radar integration, the GPM satellite bias correction, and the modeling of rainfall estimates using the XGBoost algorithm, the rainfall estimation results for the Indonesian region were obtained. Figure 9 presents the spatial rainfall estimation products for the Indonesian region. This rainfall estimate was generated in near-real time every 10 min.

We evaluated the rainfall estimation product to demonstrate the RMSE value and accuracy. Table 6 shows the results of evaluating the rainfall estimation model in this study. Furthermore, we captured and analyzed several rain events in areas with different rain patterns and variations in rainfall intensity.

**Figure 10:**
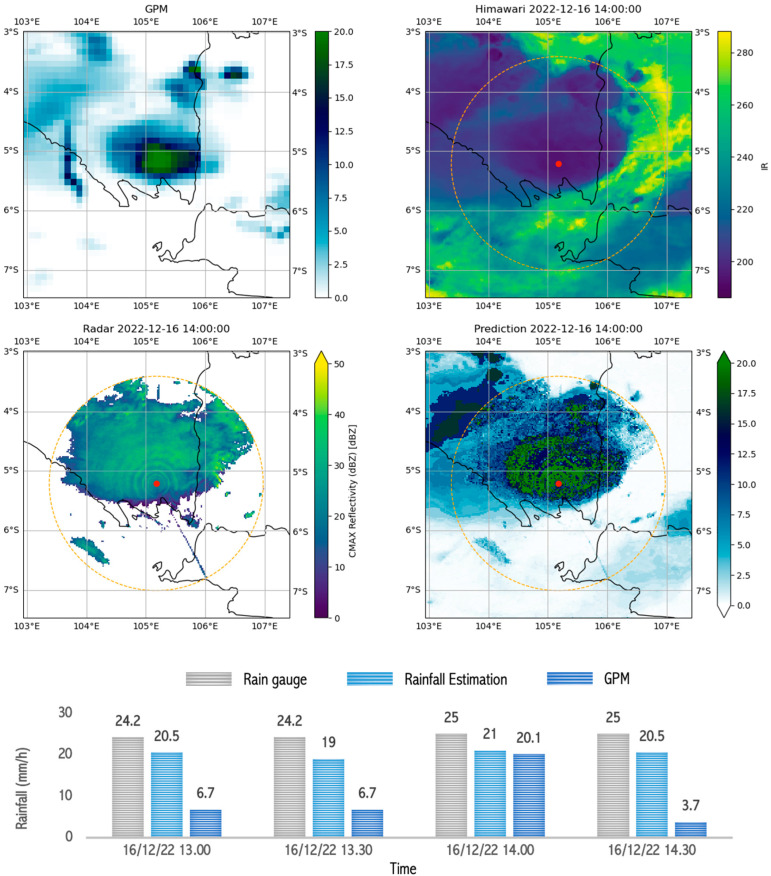
Image capture of GPM, radar, satellite, and estimation model results as well as comparison graphs of rain between rain gauge, GPM, and rainfall estimations during ongoing rain that occurred during very heavy rain (>20 mm/h) in Bandar Lampung on 16 December 2022 between 13.00 and 14.30.

Based on Figure 10, the weather radar image shows a high reflectivity (>30 dBZ), and the Himawari image shows thick clouds with a low cloud top temperature, recorded at 190 °K or −83.5 °C. This condition indicates the potential for very heavy rain in the area. At the start of the very heavy rain, the rain gauge measured 24.2 mm/h, and the model estimated rainfall to be 20.5 mm/h, while the GPM Satellite only detected normal rain, namely, 6.7 mm/h. After 1 h, all three detected very heavy rain: the rain gauge measured 25 mm/h, the rainfall model estimate was 21.0 mm/h, and the GPM satellite measurement was 20.1 mm/h. This fact shows that the estimation model is more sensitive to local variations and can quickly capture weather dynamics.

**Figure 11:**
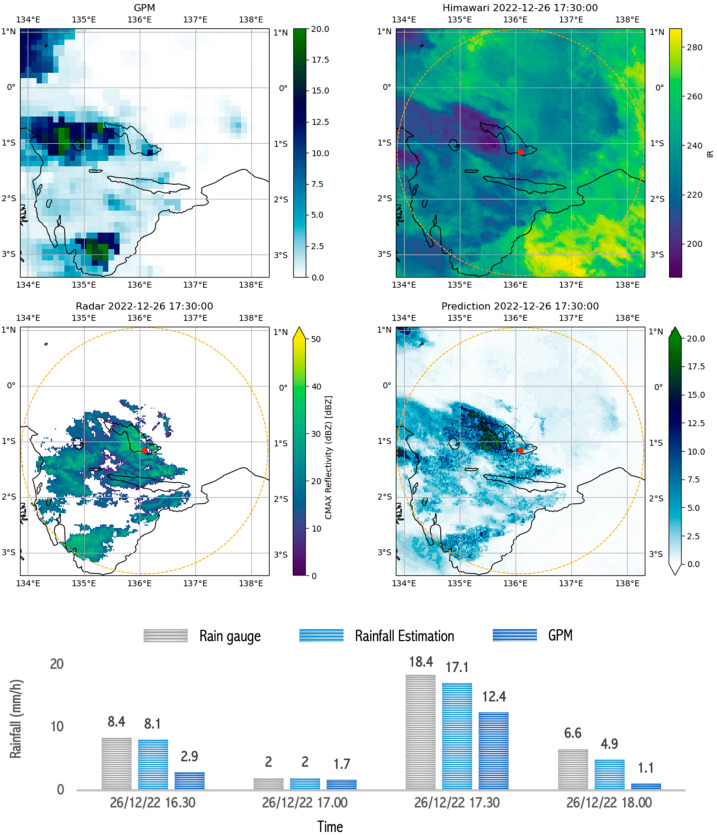
Image capture of GPM, radar, satellite, and estimation model results as well as comparison graphs of rain between rain gauge, GPM, and rainfall estimation during ongoing rain which occurred during heavy rain (10–20 mm/h) in Biak on 26 December 2022 between 16.30 and 18.00.

Figure 11 shows the Himawari satellite image features with cloud cover, which indicates the potential for heavy rainfall. The brightness temperature of the Himawari satellite was 210 °K, reflecting the presence of a lot of water vapor in the clouds. Likewise, in the radar measurements, the reflectivity of 30 dBZ indicated the number of droplets that would eventually become rain. The results of the rainfall estimation model tended to be close to the results of the rain gauge measurements; this shows its reliability. In contrast, GPM consistently recorded lower values, which may indicate a delay or underestimation.

**Figure 12:**
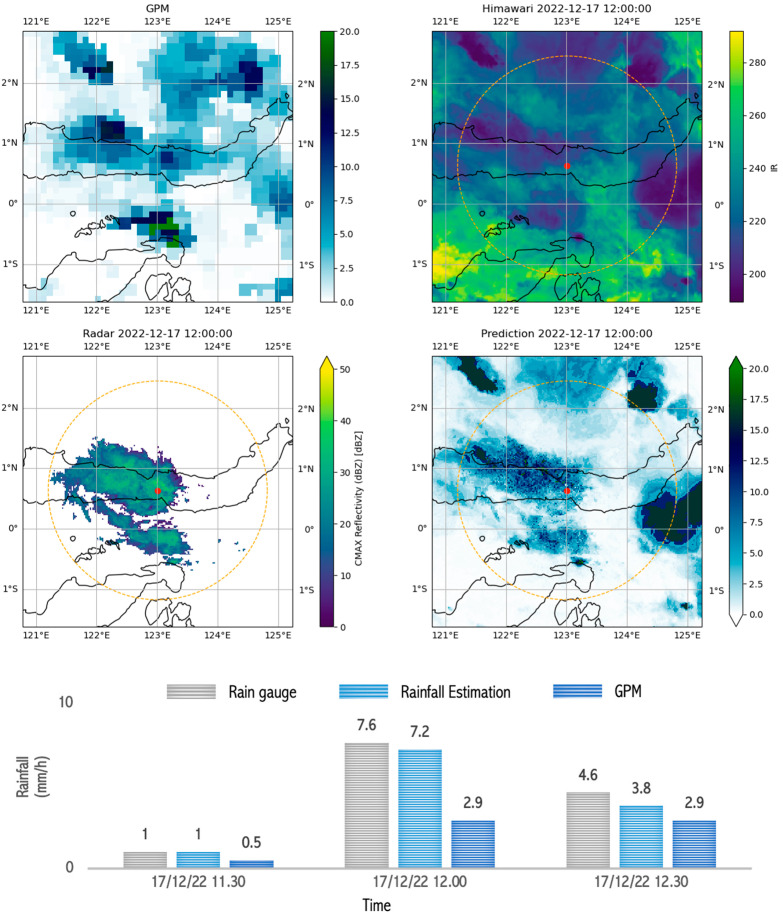
Image capture of GPM, radar, satellite, and estimation model results as well as comparison graphs of rain between rain gauge, GPM, and rainfall estimation during ongoing rain that occurred during moderate rain (10–20 mm/h) in Gorontalo on 17 December 2022 between 11.30 and 12.30.

The consistency of the model estimation results and GPM underestimation of measured rainfall was still visible in moderate rain events that occurred in Gorontalo. Figure 12 shows the rainfall estimates with images that are not too thick, indicating a relatively low rainfall intensity. The model estimated rainfall from 11:00 to 12:30 with a moderate intensity, proven to be accurate based on field observations from the rain gauge, which recorded rainfall of 1.0 mm at 11:30, increasing to 7.6 mm at 12:00, and decreasing again to 3.8 mm at 12:30. These data show that the rain only lasted a short time, around one hour, with a normal intensity, based on the estimates obtained from the rainfall model. This validation indicates that the rainfall estimation model can provide accurate rainfall projections, especially when identifying short rainfall durations.

**Figure 13:**
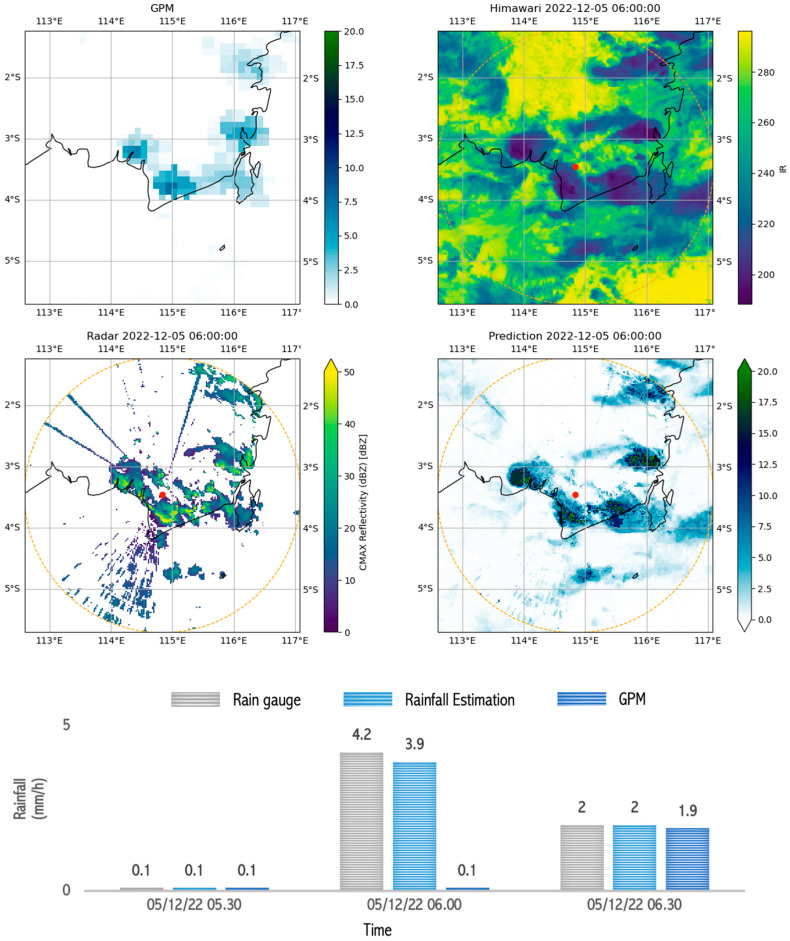
Image capture of GPM, radar, satellite, and estimation model results as well as comparison graphs of rain between rain gauge, GPM, and rainfall estimation during ongoing rain which occurred during light rain (5–10 mm/h) in Banjarmasin on 5 December 2022 between 05.30 and 06.30.

The rainfall estimates for light-rain events also showed better results when compared with the GPM rain estimates. As Figure 13 shows, the rainfall estimates based on the input Himawari data with a brightness temperature of 260 °K and radar data with a reflectivity of 15 dBZ produced a rainfall estimate of 3.9 mm/h, close to the rain gauge measurement of 4.2 mm/h. Physically, rain can, indeed, occur when the cloud top temperature approaches 260 °K [132]. However, at this temperature, the number of droplets is not very significant, as measured by the radars. Therefore, light rain occurred in a relatively short time. These estimation results reflect the model’s ability to utilize input data from radars and Himawari satellites to produce rainfall estimates that are close to observed values, including during light-rain events.

**Figure 14:**
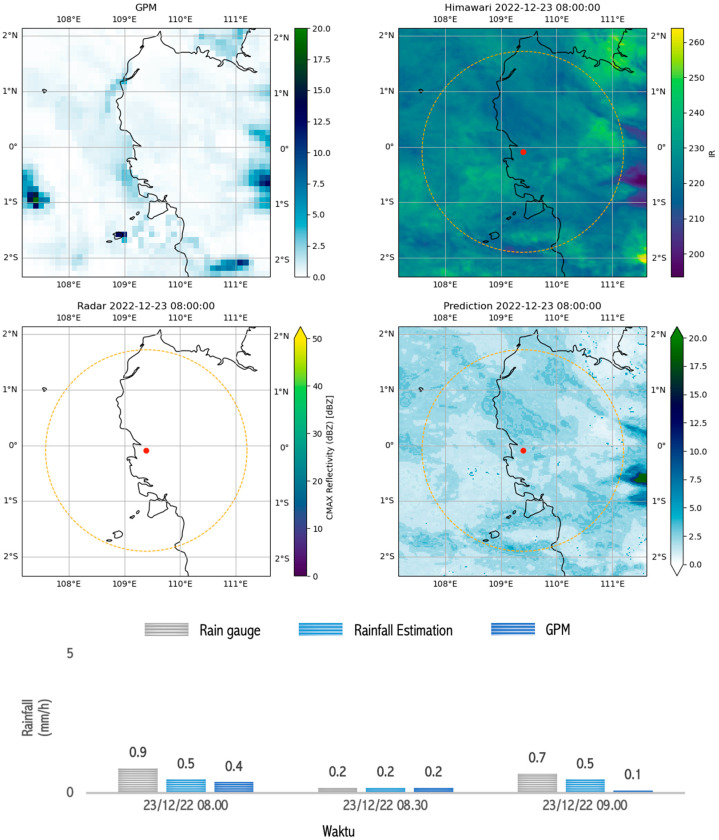
Image capture of GPM, radar, satellite, and estimation model results as well as comparison graphs of rain between rain gauge, GPM, and rainfall estimation during ongoing rain which occurred during very light rain (<1 mm/h) in Pontianak on 23 December 2022 between 08.00 and 09.00.

Very-light-rain conditions can be seen in Figure 14. The Himawari satellite data showed a brightness temperature that was quite cold, namely, 260 °K, but the radar measurements showed no reflectivity. However, very light rainfall was detected by the rain gauge. This was also detected by the model and GPM. Physically, this condition shows a lot of water vapor in the atmosphere, but very little is condensed into droplets because more is evaporating than condensing, and this condition is not measurable by radars. This could be due to the lack of aerosols in the atmosphere, which act as condensation nuclei [124]. These few droplets still become raindrops through collision and coalescence processes and fall to the Earth’s surface, even though they only become very light rain [133,134].

Based on the rainfall estimation images and the evaluation results of the rainfall estimation model, the XGBoost ensemble learning model in this research can be effectively applied to produce very accurate spatial rainfall estimates with a temporal resolution of every 10 min. Even though the training data correlation was not very good, and the data were not balanced, the XGBoost model was able to produce a low RMSE with reasonable accuracy. This demonstrates that the XGBoost model was proven to be accurate in estimating rainfall, even when it used training data, some of which had zero or no value. This is a result of the splitting process employed by XGBoost when building a decision tree during the training process. XGBoost treats non-valuable data as separate elements in the tree. If a feature has no value, the algorithm separates the data into the following two groups: one group with no value and the other group with a known value. This is the reason XGBoost does not lose information from worthless data. In fact, XGBoost is able to select the split point with the highest gain. In other words, the algorithm chooses the best way to separate data that have no value from those that have value so that the gain or profit (decrease in the loss function) is maximized. This technique is referred to as “sparsity-aware split finding” [65,135].

XGBoost can process data in parallel to handling large-scale spatial data, as well as meeting short-time resolution requirements [136]. This is known as parallel learning because XGBoost relies on an ensemble of decision trees. Each tree is built independently of the others, meaning that the building process of each tree does not depend on the results of the other trees. The data can be divided into smaller subsets, which are then processed separately. Additionally, XGBoost applies independent regularization to each tree. Therefore, each tree can be constructed separately without requiring information from the other trees. The characteristic of the independent decision tree in the XGBoost model is that it is stump-shaped, namely, it is a tree with only one level or one branch. The fact that the tree has a limited depth helps prevent overfitting and makes the tree more general and helpful in understanding the linear relationships between features and targets.

## 4. Conclusions

This study produced a model and a rainfall estimation product that use various data from different instruments, including rain gauges, weather radars, and weather satellites. In the process of model development, data preprocessing was conducted, which involved techniques such as resampling, intersection, and bilinear interpolation. Additionally, bias correction techniques were applied to the GPM satellite data, using rain gauge data as a reference. Furthermore, several weather radars were combined into an integrated weather radar network. The incorporation of the XGBoost model played a crucial role in ensuring the accuracy of rainfall estimates. Based on the discussion and analysis of the results, the rainfall estimates in this research can be applied throughout Indonesia, including areas with monsoonal, local, and equatorial rainfall patterns with near-real-time resolution, providing the latest information every 10 min. This product has the potential to accurately capture diverse rainfall patterns in Indonesia at high spatial and temporal scales. Future work will focus on estimation techniques capable of strengthening confidence levels. It not only produces a single estimation value but can also provide an overview of the uncertainty of the estimate.

## Figures and Tables

**Table 1:** The research data sources from rain gauge, weather radar, and weather satellite equipment.

Instrument	Size of Dataset	Product	Time Resolution	Spatial Resolution	Unit of Measurement
GPM satellite (NASA, USA)	9.3 GiB	Rainfall	30 min	10 × 10 km	mm/h
Himawari satellite (JMA, Japan)	1 TiB	Brightness temperature	10 min	2 × 2 km	K
Weather radars (EEC, USA)	50.7 TiB	Reflectivity	10 min	0.5 × 0.5 km	dBZ
Rain gauges (All Weather, Inc, USA)	13.3 MiB	Rainfall	1 min	Point	mm

**Table 2:** The rain gauge locations in AWOS equipment used for the bias correction of GPM rainfall products.

Location	Latitude	Longitude	Elevation (masl)
Bandar Lampung	105.174	−5.239	83
Banjarmasin	114.767	−3.439	20
Pontianak	109.402	−0.142	2
Deli Serdang	98.884	3.645	7
Gorontalo	122.852	0.638	32
Biak	136.104	−1.19	12

**Table 3:** The weather radars operating in Indonesia integrated into this study. These consisted of 35 weather radars located in various locations with diverse frequency bands, polarizations, and peak power.

No.	Location	Latitude	Longitude	Elevation (masl)	Frequency Band	Polarization	Peak Power
1	Banda Aceh	5.53	95.49	446	C	Single	250 kW
2	Nias	1.16	97.0	6	C	Single	350 kW
3	Medan	3.53	98.63	61	C	Single	250 kW
4	Padang	0.78	100.3	24	C	Single	250 kW
5	Pekanbaru	0.45	101.46	31	C	Single	250 kW
6	Bengkulu	−3.85	102.34	15	C	Single	400 kW
7	Jambi	−1.63	103.64	44	C	Single	400 kW
8	Palembang	−2.91	104.7	12	C	Single	250 kW
9	Pangkalpinang	−2.16	106.14	30	C	Single	350 kW
10	Lampung	−5.2	105.17	106	C	Single	250 kW
11	Cengkareng	−6.17	106.64	25	C	Single	250 kW
12	Pontianak	−0.08	109.39	26	C	Single	250 kW
13	Sintang	−0.04	111.45	28	C	Dual	400 kW
14	Pangkalanbun	−2.73	111.64	31	C	Single	400 kW
15	Banjarmasin	−3.46	114.84	81	C	Single	250 kW
16	Balikpapan	−1.25	116.89	50	C	Single	250 kW
17	Tarakan	3.31	117.58	45	C	Single	250 kW
18	Yogyakarta	−7.73	110.35	182	C	Single	350 kW
19	Surabaya	−7.41	112.76	3	C	Single	250 kW
20	Denpasar	−8.73	115.17	28	C	Single	250 kW
21	Lombok	−8.75	116.24	94	C	Single	400 kW
22	Bima	−8.54	118.68	45	C	Single	250 kW
23	Maumere	−8.61	122.08	36	C	Single	400 kW
24	Kupang	−10.21	123.62	326	C	Dual	400 kW
25	Majene	−3.55	118.98	30	X	Single	2 × 500 kW
26	Makassar	−4.99	119.57	11	C	Single	250 kW
27	Masamba	−2.55	120.32	66	X	Single	2 × 500 kW
28	Gorontalo	0.63	123.01	90	C	Single	250 kW
29	Ternate	0.85	127.34	105	C	Single	400 kW
30	Manado	1.5	129.91	16	C	Single	250 kW
31	Ambon	−3.71	128.09	9	C	Single	250 kW
32	Biak	−1.16	136.08	72	C	Single	250 kW
33	Sorong	−0.89	131.28	22	C	Single	250 kW
34	Timika	−4.52	136.89	54	C	Single	250 kW
35	Merauke	−8.49	131.28	88	C	Single	250 kW

**Table 4:** Hyperparameter tuning for XGBoost.

Hyperparameter	Range	Definition
learning_rate	0.01–1	The step size when updating model weights to minimize errors, which affects the speed and convergence of the training process.
max_depth	0–12	The maximum depth of a tree, which controls the complexity of the model by limiting the number of levels of splitting in each tree.
n_estimators	100–1000	The total number of decision trees to be built and used in an ensemble model, which directly affects the performance and complexity of the model.
subsample	0.1–1	The proportion of training data samples used to build each tree, introducing variation in the training process.
min_child_weight	0.1–2	The sum of instance weights required at a leaf node, which ensures that nodes will not split if they do not meet this minimum weight threshold.
gamma	0–1	The minimal reduction in loss required to split nodes, which helps control tree growth by preventing insignificant splits.
colsample_bytree	0.1–1	The proportion of features (columns) randomly selected to build each tree, which helps prevent overfitting by reducing the correlation between trees.

**Table 5:** Hyperparameter and optimal values obtained by each Bayesian optimization strategy.

Hyperparameter	Optimal Value
learning_rate	0.04
max_depth	1
n_estimators	886
subsample	0.96
min_child_weight	0.14
gamma	0.08
colsample_bytree	0.45

**Table 6:** The results of the rainfall estimation model evaluation.

Location	RMSE	Accuracy
Bandar Lampung	2.75	0.89
Banjarmasin	2.57	0.91
Pontianak	3.08	0.89
Deli Serdang	2.64	0.9
Gorontalo	1.85	0.92
Biak	2.48	0.9

## Data Availability

The data are available upon request due to restrictions.
